# Use of Acid Ceramidase and Sphingosine Kinase Inhibitors as Antiviral Compounds Against Measles Virus Infection of Lymphocytes *in vitro*

**DOI:** 10.3389/fcell.2019.00218

**Published:** 2019-10-01

**Authors:** Anika Grafen, Fabian Schumacher, Janice Chithelen, Burkhard Kleuser, Niklas Beyersdorf, Jürgen Schneider-Schaulies

**Affiliations:** ^1^Institute for Virology and Immunobiology, University of Würzburg, Würzburg, Germany; ^2^Institute of Nutritional Science, University of Potsdam, Nuthetal, Germany; ^3^Department of Molecular Biology, University of Duisburg-Essen, Essen, Germany

**Keywords:** measles virus, sphingolipids, acid ceramidase, acid ceramidase inhibitor ceranib-2, sphingosine kinase, sphingosine kinase inhibitor SKI-II

## Abstract

As structural membrane components and signaling effector molecules sphingolipids influence a plethora of host cell functions, and by doing so also the replication of viruses. Investigating the effects of various inhibitors of sphingolipid metabolism in primary human peripheral blood lymphocytes (PBL) and the human B cell line BJAB we found that not only the sphingosine kinase (SphK) inhibitor SKI-II, but also the acid ceramidase inhibitor ceranib-2 efficiently inhibited measles virus (MV) replication. Virus uptake into the target cells was not grossly altered by the two inhibitors, while titers of newly synthesized MV were reduced by approximately 1 log (90%) in PBL and 70–80% in BJAB cells. Lipidomic analyses revealed that in PBL SKI-II led to increased ceramide levels, whereas in BJAB cells ceranib-2 increased ceramides. SKI-II treatment decreased sphingosine-1-phosphate (S1P) levels in PBL and BJAB cells. Furthermore, we found that MV infection of lymphocytes induced a transient (0.5–6 h) increase in S1P, which was prevented by SKI-II. Investigating the effect of the inhibitors on the metabolic (mTORC1) activity we found that ceranib-2 reduced the phosphorylation of p70 S6K in PBL, and that both inhibitors, ceranib-2 and SKI-II, reduced the phosphorylation of p70 S6K in BJAB cells. As mTORC1 activity is required for efficient MV replication, this effect of the inhibitors is one possible antiviral mechanism. In addition, reduced intracellular S1P levels affect a number of signaling pathways and functions including Hsp90 activity, which was reported to be required for MV replication. Accordingly, we found that pharmacological inhibition of Hsp90 with the inhibitor 17-AAG strongly impaired MV replication in primary PBL. Thus, our data suggest that treatment of lymphocytes with both, acid ceramidase and SphK inhibitors, impair MV replication by affecting a number of cellular activities including mTORC1 and Hsp90, which alter the metabolic state of the cells causing a hostile environment for the virus.

## Introduction

Sphingolipids are major membrane lipid constituents and as such, their biogenesis, modifications, and turnover are tightly linked to all processes involving membrane dynamics and the cellular metabolism. Their local segregation and metabolites directly affect biophysical properties of membranes including membrane deformation, fusion, and vesicle formation, however, they also act as signal transducing molecules. Being used as signaling molecules, they govern the metabolic state and fundamental host cell responses such as apoptosis, survival, autophagy, and proliferation (Hannun and Obeid, 2008). Therefore, sphingolipids and their metabolites are potential key regulators in the life cycle of obligatory intracellular pathogens such as viruses, which rely on cellular metabolism.

Several findings revealing effects of the sphingolipid metabolism on viral infections came from animal models (Schneider-Schaulies and Schneider-Schaulies, 2015). Acid sphingomyelinase (ASM)-deficient mice are more susceptible to Sindbis virus-induced encephalomyelitis (Ng and Griffin, 2006; Ng et al., 2008), ceramides enhance the infection with Japanese encephalitis virus (Takamatsu et al., 2010), and CD8^+^ T cells of lymphocytic choriomeningitis virus (LCMV)-infected ASM-deficient mice secrete reduced levels of IFN-γ and cytotoxic granules resulting in delayed viral clearance (Herz et al., 2009). In addition, certain viruses such as major- and minor-group human rhinoviruses are able to promote the formation of ceramide-enriched membrane platforms thereby supporting their own entry into target cells (Grassme et al., 2005). Similarly, MV induces surface expression of its receptor CD150 via DC-SIGN-mediated activation of sphingomyelinases in dendritic cells, and thereby stimulates its own uptake into these cells (Avota et al., 2011). Adenovirus stimulates calcium influx and lysosomal exocytosis, a membrane repair mechanism resulting in the release of ASM and degradation of sphingomyelin (SM) to ceramides in the plasma membrane (Luisoni et al., 2015). Furthermore, SM and ASM activity appear to be involved in early steps of Ebola virus infection. In addition, the endo/lysosomal cholesterol transporter NPC1 residing in an intracellular compartment rather than at the plasma membrane serves as an entry receptor for Ebola virus (Miller et al., 2012). We found that MV brain infection in ASM-deficient mice, or after pharmacological inhibition of the ASM in mice, is enhanced due to an increased frequency of CD4^+^ Foxp3^+^ regulatory T cells (Hollmann et al., 2016). These findings highlight the potential of manipulations of the sphingolipid metabolism for novel therapeutic or immunomodulatory applications against viral infections.

In order to investigate the interference of sphingolipid metabolism with a viral infection, we used measles virus (MV) infection of lymphocytes as a model system. During the infection of a person MV first interacts with the receptor CD150 on the surface of macrophages, dendritic cells, as well as activated and memory B and T cells and is predominantly taken up by pH-independent receptor-mediated membrane fusion at the plasma membrane (Tatsuo et al., 2000; Erlenhoefer et al., 2001). Later, the virus interacts with nectin-4 to infect polarized epithelial cells (Muhlebach et al., 2011; Noyce et al., 2011). Despite the availability of an effective vaccine, measles remains a leading cause of mortality and morbidity in young children causing approximately 100,000 deaths each year worldwide (World Health Organization [WHO], 2018). The infection is associated with a number of complications including immunosuppression, and may also persist and cause subacute sclerosing panencephalitis (SSPE) (Reuter and Schneider-Schaulies, 2010; Mina et al., 2015). A specific antiviral therapy is not yet available. However, several compounds manipulating sphingolipid metabolism are available, and some of them are already in clinical use mainly applied against various forms of cancer. We wondered, if such drugs could also be used to manipulate MV replication. Vijayan et al. (2014) recently described that overexpression of the SphK-1 enhanced, and inhibition of SphK-1 (and -2) by the inhibitor SKI-II reduced MV replication in epithelial and lymphoid cell lines. They found that after MV infection of these cell lines the activity of the sphingosine kinases (SphK) and the phosphorylation of NF-kB p65 was increased. Furthermore, the NF-kB inhibitor Bay-11-7082 also reduced MV protein synthesis by preventing the phosphorylation and subsequent degradation of IkB (Vijayan et al., 2014). Accordingly, S1P-metabolizing enzymes reduced influenza virus (IAV) propagation and cytopathogenicity (Seo et al., 2010).

In the present study we investigated if some well-known inhibitors of the sphingolipid metabolism, which are partially already in clinical use, may inhibit MV replication in its primary natural target cells, i.e., CD150^+^ cells. We found that not only SKI-II (French et al., 2003; Draper et al., 2011), but also ceranib-2 (Draper et al., 2011), an inhibitor of the acid ceramidase, efficiently reduced MV replication in primary human PBL and the human B cell line BJAB. The data suggest that MV replication can be impaired by several mechanisms regulated by sphingolipid-mediated signaling pathways.

## Materials and Methods

### Cells, Viruses, and Inhibitors

All experiments involving human cells were conducted according to the principles expressed in the Declaration of Helsinki and ethically approved by the Ethics Committee of the Medical Faculty of the University of Würzburg. Primary human peripheral blood mononuclear cells (PBMCs) obtained from leuko-reduction chambers of thrombocyte donations of anonymous healthy adult volunteers were diluted 1:6 in Versene (Gibco), layered on Histopaque^®^ 1077 and purified by density gradient centrifugation. Isolated PBMCs were washed three times with (PBL) and suspended in RPMI 1640 (Gibco) medium containing 10% FBS and incubated for 2 h on plastic dishes to remove adherent monocytes. Peripheral blood lymphocytes (PBL) were collected and stimulated with phytohemagglutinin-L (PHA, 2.5 μg/ml, Roche). Stimulation was controlled by measuring CD69 expression by flow cytometry. The human B cell line BJAB was cultivated using RPMI 1640 medium containing 5% FBS. The recombinant wild-type MV rMV_IC__323_eGFP (Hashimoto et al., 2002) was propagated using Vero cells expressing CD150 (Vero-hSLAM). For the described experiments, cells were infected for 2 h, washed with PBS, and further incubated for indicated times in medium. Virus was harvested by freezing and thawing the complete cultures, thus cell-associated and supernatant virus were harvested together, and titrated using Vero-hSLAM cells. The inhibitors of SphK-1 and -2, SKI-II, of acid ceramidase, ceranib-2, of ASM, amitriptyline, of NSM, GW4869, and of Hsp90, 17-AAG, were purchased from Sigma–Aldrich. All inhibitors except amitriptyline were dissolved in dimethyl sufoxide (DMSO). The viability of cells in the presence of inhibitors was determined after 48 h incubation by flow cytometry using propidium iodide (PI; Biolegend) staining for dead cells. The percentage of dead cells in the presence of corresponding concentrations DMSO was subtracted from values obtained for various concentrations of inhibitors and the viability in the presence of 0.2% DMSO normalized to 100%.

### Antibodies and Flow Cytometry

The following primary antibodies were used in immunoblotting and flow cytometry: rabbit anti-GAPDH (Santacruz), rabbit anti-phospho-p70 S6 kinase (S6K) (Thr 389) (Cell Signaling), rabbit anti-p70 S6K (Cell Signaling), and APC-conjugated anti-human CD69 (Biolegend). Anti-human CD150 antibody clone 5C6 was generated in our laboratory (Erlenhoefer et al., 2001). The following labeled secondary antibodies were used: Alexa488-conjugated anti-mouse (Life Technology), Alexa594-conjugated anti-rabbit (Life Technology), and horseradish peroxidase (HRP)-conjugated goat anti-rabbit IgG (Cell Signaling). For flow cytometry 2 × 10^5^ cells per sample were stained with respective antibodies in FACS buffer (PBS containing 0.4% bovine serum albumin (BSA) and 0.02% sodium azide). Dead cells were stained with PI (Biolegend). Cells were acquired immediately using a LSR II flow cytometer (BD) and the data were analyzed using FlowJo (Cytek Development) software.

### Virus Uptake Assay

To measure virus uptake, cells were pretreated with increasing concentrations of inhibitors as indicated for 1 h prior to infection with MV (MOI = 0.5 for primary PBL and MOI = 0.1 for BJAB cells) for 2 h at 37°C. Then the cells were washed with PBS and further incubated in medium for 22 h at 37°C to allow viral transcription and protein expression. The percentage of infected (viral eGFP-positive) cells was then determined by flow cytometry (not the mean fluorescence intensity which reflects the amount of viral protein expression).

### SDS-PAGE and Immunoblotting

Cells (5 × 10^6^) were lysed at 4°C for 1 h in 1 ml of lysis buffer [50 mM Tris–HCl, pH 8.0, 150 mM sodium chloride (NaCl), 1.0% Igepal CA-630 (NP-40), 0.5% sodium deoxycholate, 0.1% sodium dodecyl sulfate (SDS)] containing complete protease inhibitor cocktail (Sigma–Aldrich) and 1 mM DL-dithiothreitol (DTT). The protein quantification was done using the bicinchoninic acid (BCA) assay. An equal amount of proteins was heated at 95°C for 5 min in reducing sample buffer (50 mM Tris–HCl, pH 6.8, 2% SDS, 10% glycerol, 1% β-mercaptoethanol, 12.5 mM ethylenediaminetetraacetic acid (EDTA), 0.02% bromophenol blue) and applied to 12% SDS-polyacrylamide gel electrophoresis. Proteins were blotted semidry on nitrocellulose membranes (Amersham) followed by blocking with 5% dry milk (AppliChem) or 5% BSA in Tris-buffered saline with 0.05% Tween-20. The membranes were then incubated with specific primary antibodies and HRP-conjugated secondary antibodies. Signals were visualized using Chemiluminescent FemtoMax^TM^ Super Sensitive HRP Substrate (Rockland). The densitometric quantification of protein bands of target proteins and respective housekeeping gene were done using Li-Cor Odyssey Pc imaging System Image Studio Version 4.0 (Li-Cor Biosciences). The fold changes in target proteins were normalized to band densities of respective GAPDH and fold changes in phosphorylated proteins were normalized to the band densities of total protein or GAPDH levels. All western blotting experiments were repeated at least three times and representative images are shown.

### Lipid Analysis

For lipid analysis 8 × 10^6^ PBL or 1 × 10^6^ BJAB cells per sample were resuspended in 500 μl methanol and subsequently subjected to lipid extraction using methanol/chloroform (2:1, v:v) (Gulbins et al., 2018). The extraction solvent contained d_7_-Sph, d_7_-sphingosine-1-phosphate (d_7_-S1P), C17-ceramide, and C16-d_31_-SM (all Avanti Polar Lipids, Alabaster, United States) as internal standards. Sample analysis was carried out by liquid chromatography tandem–mass spectrometry (LC–MS/MS) using either a TQ 6490 mass spectrometer (for Sph and S1P) or a QTOF 6530 mass spectrometer (for ceramides and SMs) (Agilent Technologies) operating in the positive electrospray ionization mode (ESI+). The following selected reaction monitoring (SRM) transitions were used for quantification: *m/z* 300.3 → 282.3 for Sph, *m/z* 380.3 → 264.3 for S1P, *m/z* 307.3 → 289.3 for d_7_-Sph, and *m/z* 387.3 → 271.3 for d_7_-S1P. The precursor ions of ceramide or SM species (differing in their fatty acid chain lengths) were cleaved into the fragment ions *m/z* 264.270 or *m/z* 184.074, respectively (Kachler et al., 2017). Quantification was performed with Mass Hunter Software (Agilent Technologies).

### Statistical Analysis

Statistical analysis was performed using Microsoft Excel or GraphPad Prism 6. Two groups were analyzed using unpaired two-tailed Student’s *t*-test and more than two groups were analyzed with one-way ANOVA. *P*-values lower than or equal to 0.05 were considered statistically significant (^∗^*P* ≤ 0.05, ^∗∗^*P* ≤ 0.01, ^∗∗∗^*P* ≤ 0.001). The data represent mean ± SD of at least three independent experiments.

## Results

### The Sphingosine Kinase Inhibitor SKI-II Inhibits MV Replication in Primary Human PBL

**FIGURE 1:**
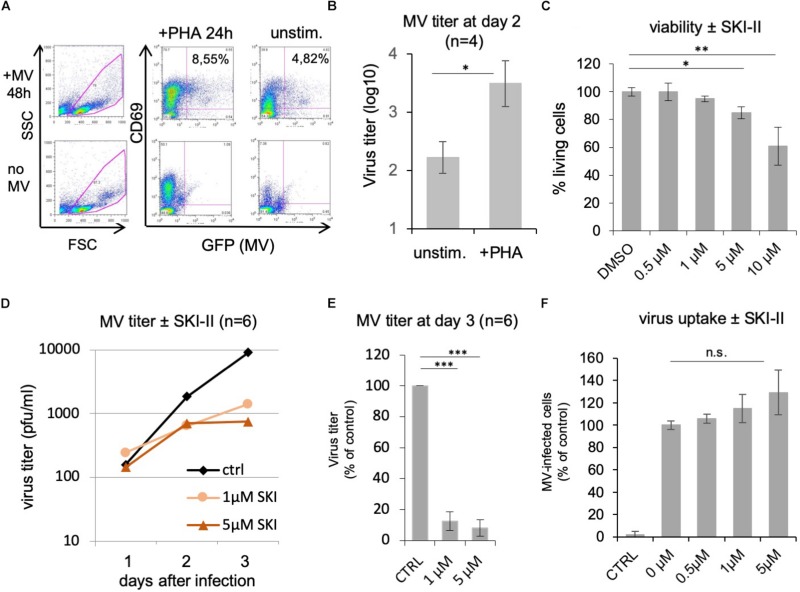
SKI-II inhibits MV replication in primary human PBL. The stimulation, infection, and viability of the PBL without and with PHA (2.5 μg/ml) for 24 h was controlled by flow cytometry measuring the expression of CD69, viral eGFP, and propidium iodide (PI). An example of infected and uninfected PBL in the presence and absence of PHA is shown in panel **(A)**. Panel **(B)** shows a comparison of the MV titer produced by unstimulated and PHA-stimulated PBL (1 × 10^6^ cells) as determined at day 2 after infection at a MOI of 0.1 (*n* = 4; with ^∗^*P* ≤ 0.05) using Vero-hSLAM cells for titration. **(C)** PI incorporation assay as control for the viability of the cells. PHA-stimulated PBL were treated for 48 h with SKI-II as indicated, dead cells were stained with PI, and percentages of living cells determined by flow cytometry (normalized to DMSO control: 100%). **(D)** Primary human PBL were stimulated with PHA and 1 h pretreated with 0.2% DMSO as mock-treated control (ctrl) or 1 and 5 μM SKI-II prior to infection with MV (MOI = 0.1). Newly synthesized infectious virus (cell bound plus supernatant) was titrated using Vero-hSLAM cells 1, 2, and 3 days after infection (*n* = 6 with PBL from six independent blood donors). **(E)** Virus titers at day 3 after infection in the presence of SKI-II (same data as in panel **D**) were significantly reduced (Student’s *t-*test with ^∗∗∗^*P* < 0.001) and are presented as percentage of mock-treated control. **(F)** To measure the virus uptake by PBL, cells were pretreated with 0.2% DMSO (=0 μM inhibitor) or increasing concentrations of SKI-II as indicated for 1 h prior to infection with MV (MOI = 0.5). The percentage of infected eGFP-positive cells was quantified by flow cytometry 24 h after infection and is presented as percentage of DMSO (=0 μM inhibitor) control. CTRL is the negative control without virus. ^∗∗^*P* ≤ 0.01.

Peripheral blood lymphocytes from healthy donors were stimulated with PHA for 24 h prior to treatment with inhibitors and infection with MV. Their activation, infection, and viability in the presence of inhibitors were controlled by flow cytometry (Figures 1A–C). A representative control showing 24 h PHA-stimulated PBL, which were subsequently infected for 48 h with MV at a MOI of 0.1 is given in Figure 1A. We were using PHA-stimulated PBL since stimulation increases the titer of newly synthesized MV approximately 20-fold (Figure 1B). The viability of PHA-stimulated PBL was determined using PI in the presence of increasing concentrations of SKI-II (Figure 1C). In further experiments we used 1 and 5 μM SKI-II, concentrations at which ≥95 and ≥85%, respectively, of PBL were viable.

To determine the effect of SphK inhibition on MV replication, PBL from six healthy donors were infected with MV at a MOI of 0.1 in the absence and presence of 0, 1, and 5 μM of SKI-II, and the newly synthesized MV (cell bound plus supernatant) was prepared and titrated after 1, 2, and 3 days (Figure 1D). The viral titers were similar at day 1, and reduced 2 and 3 days after infection. At day 3 the reduction was approximately 1 log (90%). Results at day 3 are presented as percentage of control cells with significances (Figure 1E). In order to investigate if viral entry into PBL is affected by SKI-II, we first measured if expression of the MV receptor CD150 is altered by SKI-II. This was not the case after 1, 24, and 48 h of treatment with 1 and 5 μM of the inhibitor (not shown). Then we did a virus uptake assay and quantified the percentage of infected (viral eGFP-positive) cells at day 1 after infection by flow cytometry. The percentage of infected cells (and thus virus uptake) was not reduced by SKI-II, but there was rather a tendency (not significant) toward increased virus uptake (Figure 1F). These results show that virus uptake is not impaired by SKI-II, but that intracellular virus replication is reduced later (at days 2 and 3) after infection.

### Effects of Other Inhibitors of the Sphingolipid Metabolism

**FIGURE 2:**
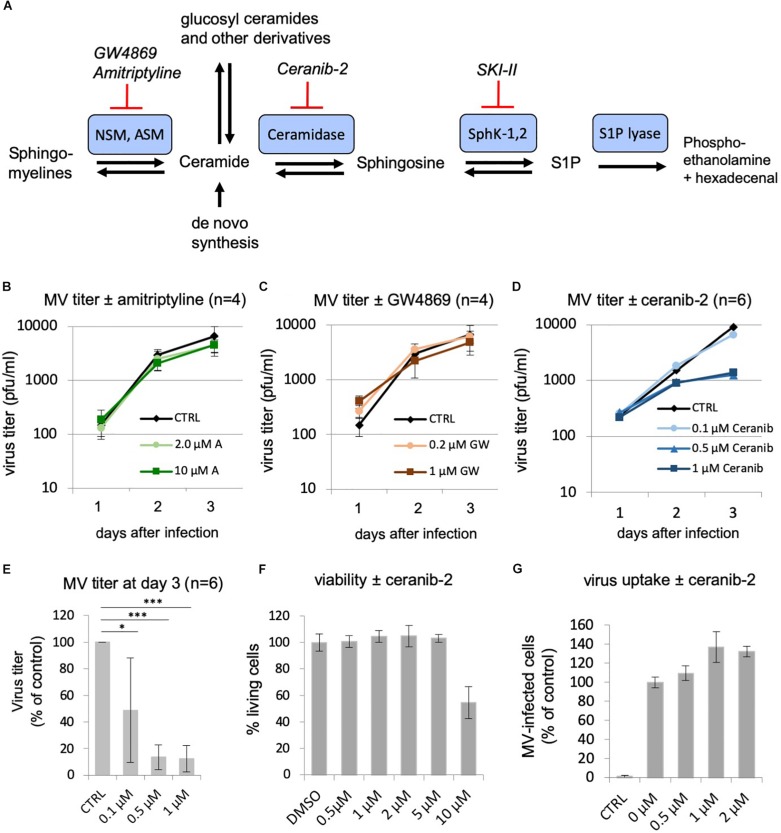
Effects of other inhibitors of the sphingolipid metabolism: inhibition of MV replication by the acid ceramidase inhibitor ceranib-2. **(A)** Schematic representation of a part of the sphingolipid metabolism with involved enzymes (the biosynthesis pathway of ceramides from serine and palmitoyl-CoA and modification pathways to ceramide-1-phosphate and glucosyl-ceramides and complex glycosphingolipids are not shown). NSM, neutral sphingomyelinase; ASM, acid sphingomyelinase; SKI-II, sphingosine kinase inhibitor 2; S1P, sphingosine-1-phosphate. **(B–D)** Primary human PBL were stimulated with PHA (2.5 μg/ml) for 24 h and pretreated with DMSO vehicle (CTRL) or increasing concentrations of inhibitor 1 h prior to infection with MV (MOI = 0.1). Newly synthesized infectious virus (cell bound plus supernatant) was titrated using Vero-hSLAM cells 1, 2, and 3 days after infection. PBL were treated with amitriptyline (2 and 10 μM; *n* = 4; **B**), GW4869 (0.2 and 1.0 μM; *n* = 4; **C**), and ceranib-2 (0.1, 0.5, and 1 μM; *n* = 6 with PBL from independent blood donors; **D**). **(E)** Virus titers at day 3 after infection in the presence of ceranib-2 are significantly reduced. Standard deviations are shown. The statistical evaluation was done using Student’s *t*-test. **(F)** The viability of the PBL was controlled using propidium iodide incorporation assay. **(G)** To measure virus uptake by PBL, cells were pretreated with increasing concentrations of ceranib-2 as indicated for 1 h prior to infection with MV (MOI = 0.5). The percentage of infected eGFP-positive cells was quantified by flow cytometry 24 h after infection and is presented as percentage of DMSO (=0 μM inhibitor) control. CTRL is the negative control without virus.

Having observed this inhibition of MV replication by the inhibitor of the SphKs, we wondered if inhibition of other enzymes of the sphingolipid metabolism may also influence virus replication. While inhibition of the sphingomyelinases is supposed to reduce cellular ceramide content, inhibition of the ceramidase should increase the amount of ceramide in the cell. We therefore investigated the effects of the well-established inhibitors of acid and neutral sphingomyelinases, amitriptyline, and GW4869, respectively, and of the acid ceramidase, ceranib-2 (Figure 2A). The toxicity of the inhibitors was determined and the concentrations used accordingly (Figure 2F; not shown for amitriptyline and GW4869). Analyzing the effects on MV replication in PBL, we found that amitriptyline and GW4869 had no inhibitory effect (Figures 2B,C). Interestingly, GW4869 even had a replication stimulating effect within the first 24 h after infection, which at days 2 and 3 after infection was not detected any more. In contrast to the sphingomyelinase inhibitors, ceranib-2 inhibited MV replication at days 2 and 3 after infection (Figure 2D). At day 3 after infection, 0.5 and 1 μM ceranib-2 inhibited MV replication in PBL by approximately 90% (Figure 2E). The viability of the PBL was not affected at these concentrations of ceranib-2 (Figure 2F). CD150 expression was not altered in dependency of ceranib-2 after 1, 24, and 48 h of treatment (not shown). Determining virus uptake in the presence of ceranib-2 we found, similar as with the inhibitor SKI-II, that the percentage of MV-infected cells 24 h after infection was not reduced, but rather slightly increased with increasing concentrations of ceranib-2 (Figure 2G). Thus, inhibition of the acid ceramidase in primary human PBL had very similar effects on MV replication as inhibition of SphKs.

### Sphingolipid Composition in PBL After Treatment With the Inhibitors Ceranib-2 and SKI-II

**FIGURE 3:**
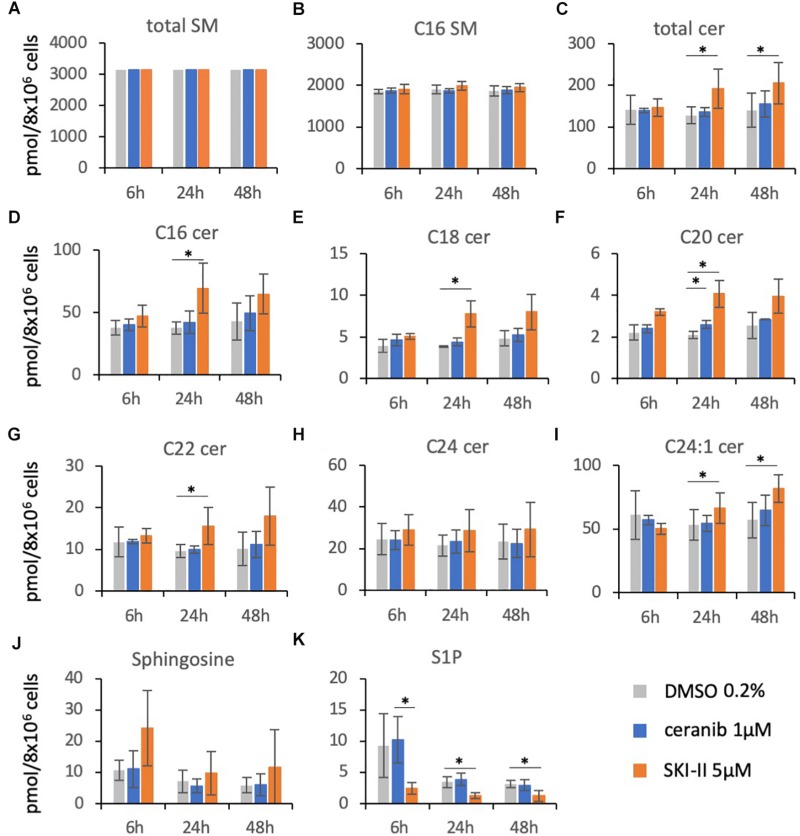
Sphingolipid composition in primary human PBL after treatment with the inhibitors ceranib-2 and SKI-II. PBL were treated for 6, 24, and 48 h with DMSO 0.2% as vehicle control (gray), or 1 μM ceranib-2 (blue) or 5 μM SKI-II (orange). The samples (8 × 10^6^ cells per sample) were processed for analysis of sphingomyelins (total SM and C16 SM), ceramides (total Cer, C16, C18, C20, C22, C24, C24:1), sphingosine, and S1P by mass spectrometry. Results from PBL of three independent donors (*n* = 3) were normalized to the mean amount of total SM measured **(A)** and are presented as pmol/sample, as indicated **(A–K)**. ^∗^ represents significant differences calculated by Student’s *t*-test (*n* = 3).

Based on their similar effects, the question arises of whether both inhibitors (SKI-II and ceranib-2) might act via similar or different mechanisms on MV replication, and which sphingolipids might potentially act as signaling molecules. To investigate the inhibitor effects on the sphingolipid rheostat, we treated PHA-stimulated PBL with these inhibitors and quantified SMs, ceramides, Sph, and S1P by LC–MS/MS (Figure 3). For the analysis we chose 1 μM ceranib-2 and 5 μM SKI-II, because these concentrations led to good inhibition of MV infection. Ceranib-2 had no significant effect on SM and ceramide levels except ceramide C20 which was slightly increased at 24 h (Figure 3F). Surprisingly, 5 μM SKI-II caused significant increases in total ceramide and C16, C18, C20, C22, and C24:1 ceramides. As expected, SKI-II treatment reduced S1P concentrations in PBL (Figure 3K). Likewise, there was a tendency that SKI-II led to increased Sph concentrations, which however was not significant due to the high variability between blood donors (Figure 3J).

### SKI-II and Ceranib-2 Inhibit MV Replication in BJAB Cells

**FIGURE 4:**
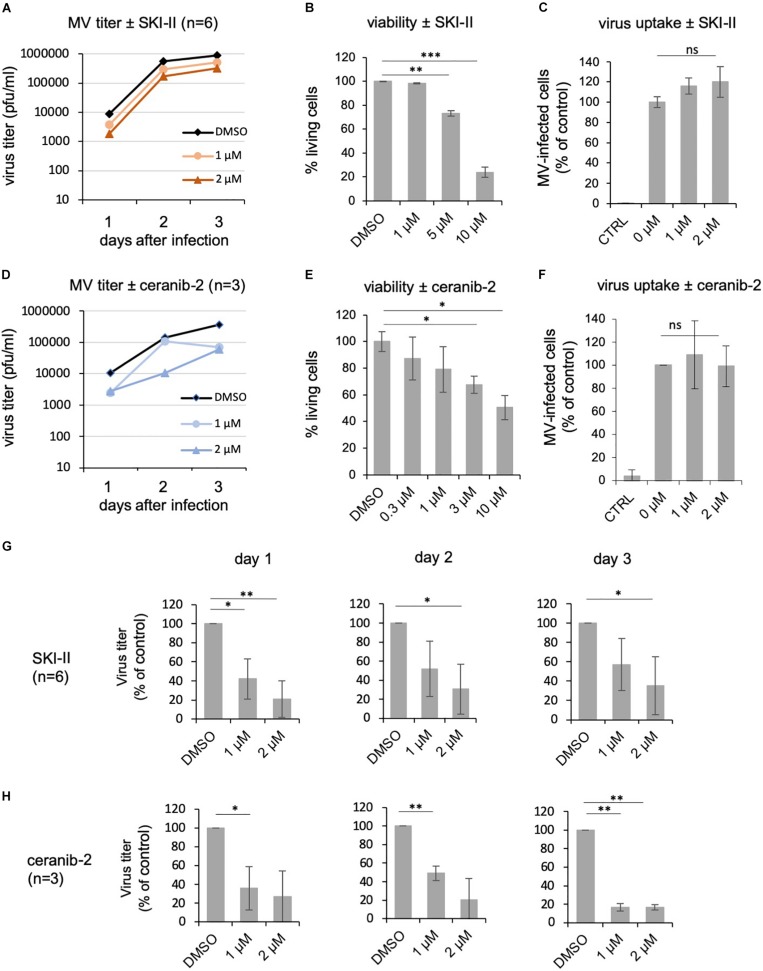
SKI-II and ceranib-2 inhibit MV replication in BJAB cells. BJAB cells were pretreated with DMSO 0.2% as vehicle control or 1 and 2 μM SKI-II **(A)** and ceranib-2 **(D)**, prior to infection with MV (MOI = 0.1), and newly synthesized virus was titrated using Vero-hSLAM cells. The viability of the BJAB cells was determined by propidium iodide incorporation by FACS in the presence of increasing concentrations of SKI-II **(B)** and ceranib-2 **(E)**. To determine the effect of the inhibitors on virus uptake, BJAB cells were treated with inhibitors for 1 h prior to infection with MV (MOI = 0.1). The percentage of infected eGFP-positive BJAB cells in the presence of increasing concentrations of SKI-II **(C)** and ceranib-2 **(F)** was quantified by flow cytometry 24 h after infection and is presented as percent of DMSO control (=0 μM inhibitor). The reductions in viral titers by SKI-II and ceranib-2 (same data as in panels **A,D**) are shown separately for days 1, 2, and 3 after infection and after treatment of cells with SKI-II **(G)** and ceranib-2 **(H)** to demonstrate more clearly the inhibitor effects at different days and to show variations with significances. The statistical evaluation was done using the Student’s *t*-test.

Because variations between primary PBL from different donors may have hampered the achievement of more significant results, we decided to use a human B cells line, BJAB, to analyze effects of the inhibitors. However, before doing so, we confirmed that both inhibitors reduce MV replication similarly in BJAB cells as in primary PBL. Treatment of BJAB cells with 1 or 2 μM SKI-II led to a dose-dependent reduction of newly synthesized virus at days 1, 2, and 3 after infection with MV (Figure 4A). Higher concentrations of SKI-II were not used because BJAB cells were more sensitive to SKI-II than primary PBL and approximately 80% died in the presence of 10 μM SKI-II (Figure 4B). In contrast to primary PBL (Figure 1D), the titration of newly synthesized virus from infected BJAB cells showed that SKI-II reduced viral titers already within the first 24 h after infection (Figures 4A,G), while this reduction was generally not as pronounced as in primary cells (Figures 1A,E). Investigating viral uptake, similarly as in PBL (Figure 1F) and in spite of the titer reduction, the percentage of infected cells 24 h after infection was not reduced with increasing concentrations of SKI-II (Figure 4C). Thus, virus uptake was not affected by SKI-II, but intracellular viral replication was inhibited.

Using the acid ceramidase inhibitor, there was a clear reduction of newly synthesized viral titers in the presence of 1 or 2 μM ceranib-2 (Figure 4D). Higher concentrations of ceranib-2 (3–10 μM) led to a reduction of the viability (Figure 4E). Virus uptake was not affected by 1 or 2 μM ceranib-2 (Figure 4F). As observed with SKI-II, MV replication was reduced by ceranib-2 in BJAB cells already at day 1. Inhibition was, however, more pronounced at day 3 (Figure 4H). Taken together, both inhibitors impaired MV replication in BJAB cells similarly as observed in primary PBL, although the inhibitory effects emerged faster in BJAB cells being detected already at day 1 after infection.

### Sphingolipid Composition in BJAB Cells Treated With Inhibitors in the Presence and Absence of MV Infection

**FIGURE 5:**
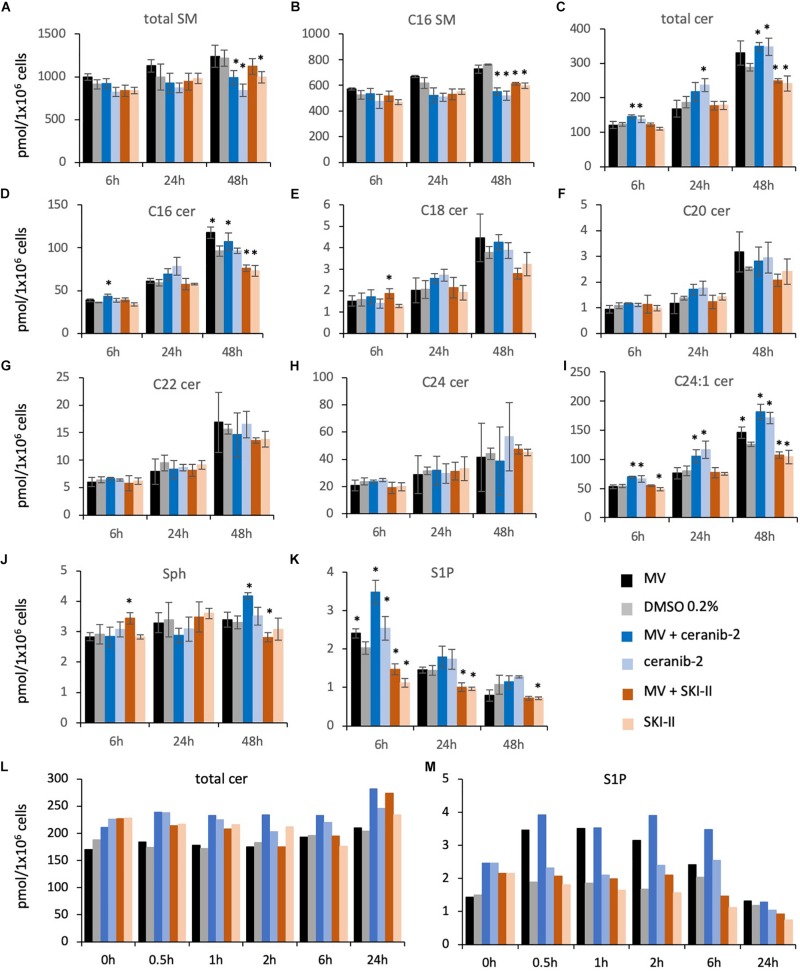
Sphingolipid composition in BJAB cells after treatment with the inhibitors ceranib-2 and SKI-II and infection with MV. The sphingolipid composition was determined in MV-infected (black bars; MOI = 0.5) and uninfected control (0.2% DMSO)-treated (gray bars) BJAB cells (1 × 10^6^ per sample), in 3 μM ceranib-2-treated infected (dark blue) and uninfected (light blue), and in 3 μM SKI-II-treated infected (dark orange) and uninfected (light orange) BJAB cells. Results for total sphingomyelins (SM), C16 SM, ceramides (total Cer, C16, C18, C20, C22, C24, C24:1), sphingosine (Sph), and S1P are presented as pmol/sample per 1 × 10^6^ cells as indicated **(A–M)**. Stars in panels **A–K** designate significant (^∗^*P* ≤ 0.05) differences in comparison to control (0.2% DMSO-treated) cells (*n* = 3; Student’s *t*-test). In panels **L,M** (*n* = 1), the significances between treatment groups were calculated by paired *T*-test and one-way ANOVA. In panel **L**, the DMSO control values (0–6 h) were significantly different from MV + ceranib-2- and ceranib-2-treated cell values (*P* = 0.001 and *P* = 0.002, respectively). In panel **M**, the DMSO control values were significantly different from MV and MV + ceranib-2-treated cell values (*P* = 0.04 and *P* = 0.007, respectively).

For the sphingolipid analysis in BJAB cells we chose 3 μM ceranib-2 in order to see clearer effect on ceramide levels than in PBL, and 3 μM SKI-II. Treatment of the cells with ceranib-2 led to reduced levels of total SMs and C16 SM at 48 h in the presence or absence of infection (dark and light blue bars) in comparison to the DMSO control (gray bar; Figure 5A), while total ceramide levels were increased after 6, 24, and 48 h (Figure 5C). Increased ceramide levels were found for total ceramide, C16, and C24:1, but not for C18, C20, C22, and C24 ceramides. Interestingly, MV infection and ceranib-2 treatment led to increased S1P levels at 6 h (Figure 5K). SKI-II treatment led to reduced total SM, C16 SM levels, total ceramide, C16, and C24: levels at 48 h in the absence and presence of infection (Figures 5A–I; dark and light orange bars). In addition, Sph was increased at 6 h after infection (Figure 5J), while the S1P concentrations were decreased by SKI-II at 6, 24, and 48 h (Figure 5K).

In order to have a closer look at the MV-induced increase of S1P at early times after infection (Figure 5K), we did an additional experiment analyzing sphingolipids at 0.5, 1, 2, 6, and 24 h after infection in the presence and absence of inhibitors. Here we observed increased total ceramide concentrations shortly after treatment with the inhibitors (Figure 5L), and a strong S1P increase after 0.5–6 h after MV infection also in the presence of ceranib-2, which, however, was not observed in the presence of SKI-II (Figure 5M). Thus, SKI-II prevented the transient increase of S1P induced by the MV infection. In summary these data in BJAB cells showed that ceranib-2 led to clearly increased ceramide levels, while SKI-II led to decreased levels of S1P and some ceramide species. The data suggest that in these cells ceranib-2 may act antivirally via the increase in ceramide concentrations, whereas SKI-II may predominantly act antivirally via the reduction of S1P concentrations.

### Reduced Metabolic Activity in Inhibitor-Treated Lymphocytes Affects MV Replication

**FIGURE 6:**
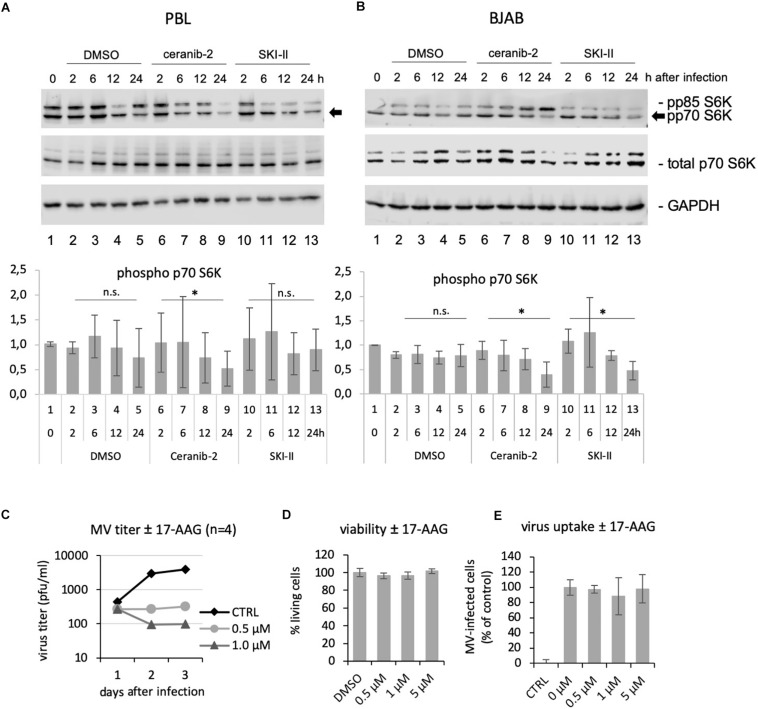
Ceranib-2 and SKI-II-mediated reduction of p70 S6K phosphorylation, and inhibition of MV replication by the Hsp90 inhibitor 17-AAG. Western blots from cell extracts of human primary PBL **(A)** and BJAB cells **(B)** which were treated with 0.2% DMSO as control, 3 μM ceranib-2, and 5 μM SKI-II for 2, 6, 12, and 24 h as indicated were developed with antibodies against phosphorylated p70 S6K (also detecting p85), total p70 S6K, and GAPDH. Quantifications of phosphorylated p70 S6K related to GAPDH of three blots of PBL from independent blood donors and BJAB of independent experiments are shown. **(C)** PBL were treated with increasing concentrations of the Hsp90 inhibitor 17-AAG, infected with MV at a MOI of 0.1, and newly synthesized virus titrated after 1, 2, and 3 days using Vero-hSLAM cells (*n* = 4). **(D)** Viability and virus uptake **(E)** was assessed as described (*n* = 3).

One common effect of increased ceramide concentrations as well as decreased S1P concentrations is to impair cell metabolism and mTORC1 activity (Dobrowsky et al., 1993; Mukhopadhyay et al., 2009; Apostolidis et al., 2016), while efficient MV replication in PBL depends on mTORC1 activity (Tiwarekar et al., 2018). We therefore investigated if the inhibitors ceranib-2 and/or SKI-II may lead to reduced mTORC1 activity. We used the phosphorylation of p70 S6K as marker for mTORC1 activity. We found that ceranib-2 reduced the phosphorylation of p70 S6K in comparison to DMSO-treated controls in PBL, and that both inhibitors, ceranib-2 and SKI-II, reduced the phosphorylation of p70 S6K in BJAB cells (Figures 6A,B). These results suggest that both inhibitors act at least by one common pathway reducing the metabolic activity of the cells, which reduces the capacity to replicate MV.

In addition, it is known that S1P also targets and activates chaperones such as GRP94 and Hsp90, which among other effects enable NF-kB activation (Xia et al., 2002; Alvarez et al., 2010; Park et al., 2015, 2016), and that Hsp90 activity is required for the replication of a number of viruses including MV (Connor et al., 2007; Smith et al., 2010; Bloyet et al., 2016; Katoh et al., 2017). Therefore, we tested the effect of the Hsp90 inhibitor 17-AAG in primary human PBL. Treatment of PBL with 0.5 and 1 μM 17-AAG strongly reduced viral titers 2 and 3 days after infection, but not yet at day 1 after infection (Figure 6C). This observed kinetic of inhibition was similar to that observed with the inhibitors SKI-II (Figure 1D) and ceranib-2 (Figure 2D) in PBL. The viability and virus uptake were not affected by 17-AAG (Figures 6D,E).

## Discussion

The two inhibitors ceranib-2 and SKI-II have been described as compounds with excellent potential for development as new anticancer drugs (French et al., 2003; Draper et al., 2011; Aurelio et al., 2016). SphKs are upregulated in many cancers and thought to play a key role in disease progression through increases in S1P and decreases in ceramide and Sph levels, promoting tumor growth and survival (Santos and Lynch, 2015). Acid ceramidase is highly upregulated in breast tumors and treatment with ceranib-2 significantly induced apoptosis in human breast cancer cell lines (Vethakanraj et al., 2018). In mice, ceranib-2 was found to delay tumor growth in a syngeneic tumor model without hematologic suppression or overt signs of toxicity (Draper et al., 2011). In contrast to cancer cells, normal primary cells are less prone to induction of apoptosis and cell death upon treatment with such inhibitors. This led us to the hypothesis that these inhibitors could eventually be applied against viral infections without damaging primary cells including antiviral lymphocytes. Indeed, we found that already relatively low concentrations of SKI-II (1 μM) and ceranib-2 (0.5 μM) reduced MV replication in primary PBL, while the expression of the MV receptor CD150 and viral uptake was not altered.

That increased ceramide can have an antiviral role has been described earlier for various viruses such as HIV (Finnegan et al., 2004), hepatitis B virus (HBV) (Tatematsu et al., 2011), Dengue virus (Perera et al., 2012), and IAV (Hidari et al., 2006; Tafesse et al., 2013; Soudani et al., 2019). In the case of HIV, this was mediated through a reduction in virus entry and perturbation of host cell membrane structure leading to the production of non-infectious viral progeny, and in the case of IAV through impaired glycoprotein transport and maturation of viral particles. However, on the other hand, sphingolipids including ceramides may also be supportive for virus replication as in the case of hepatitis C and West Nile virus (Zhang et al., 2019). Our sphingolipid analyses after treatment of cells with SKI-II and ceranib-2 led to different results for PBL and BJAB cells. In PBL, SKI-II increased the concentrations of some ceramides and ceranib-2 had no significant effect. In BJAB cells, SKI-II decreased the concentration of some ceramides, while ceranib-2 led to an increase. Furthermore, SKI-II reduced S1P concentrations in PBL and BJAB cells. Thus, because of these findings and because intracellular signaling effects of ceramides and S1P may be locally limited events (for example at lysosomes or the endoplasmic reticulum), these results unfortunately did not allow clear conclusions. Interestingly, both, increased ceramide as well as decreased S1P concentrations, reduce the mTORC activity as it is known that ceramide acts on the SET protein and decreases mTORC activity via the phosphatase PP2A (Dobrowsky et al., 1993; Li et al., 1996; Neviani et al., 2005; Trotta et al., 2007; Mukhopadhyay et al., 2009; Shi, 2009; Oaks and Ogretmen, 2014) and that S1P affects mTORC activity via Hsp90 and raptor (Delgoffe et al., 2009). As demonstrated for regulatory T cells, dephosphorylation by PP2A inactivates mTORC1 and thus reduces the cell metabolism including protein translation and lipid synthesis (Apostolidis et al., 2016). Among others, mTORC1 regulates the ribosomal S6 protein kinase-1, of which due to alternative translation two isoforms are known in mammalian cells, p85 and p70. The mTOR kinase phosphorylates and thereby activates p70 at T389 and p85 at T412 (Ruvinsky and Meyuhas, 2006). Now we found that treatment of cells with ceranib-2 reduced the phosphorylation of p70 S6K in PBL and that SKI-II reduced p70 S6K phosphorylation in PBL and BJAB cells indicating reduced mTORC1 activity. Because MV replication requires mTORC activity (Tiwarekar et al., 2018), it is likely that this is a common effect of the inhibitors affecting MV replication. We cannot exclude other (side-)effects of the inhibitors; however, the main point of the paper remains that the inhibitors affect MV replication. The finding suggests that the inhibitor-induced reduction of the MV replication is at least partially due to this reduction in mTORC1 activity.

We also found that MV infection led to a strong S1P increase between 0.5 and 6 h after infection, which was prevented by SKI-II and thus a potential S1P anti-apoptotic/pro-survival signal that may support MV replication is not provided. S1P and SphK1 were demonstrated to signal intracellularly via interaction with TRAF2, RIP1 to activate Hsp90 and NF-κB (Xia et al., 2002; Alvarez et al., 2010; Park et al., 2015, 2016). Thus, SKI-II prevents a signal that the virus itself induces in order to support its own replication. Interestingly, the chaperone Hsp90 is required for the function of polymerases of many viruses including MV (Connor et al., 2007; Smith et al., 2010; Bloyet et al., 2016; Katoh et al., 2017). In Vero and HeLa cells and brain slices of MV-susceptible mice it has been observed that Hsp90 inhibition impairs MV replication (Bloyet et al., 2016). Inhibition or lack of Hsp90 is preventing the proper folding of newly synthesized viral polymerases and facilitates their degradation (Bloyet et al., 2016). Because S1P regulates Hsp90 activity, it is likely that the SKI-II-mediated decrease in S1P concentrations also impairs Hsp90 in PBL, which may contribute to the observed inhibition of MV replication. We showed here that Hsp90 inhibition in PBL strongly reduced MV replication at days 2 and 3 after infection, but did not affect MV replication at day 1. This kinetic of inhibition is typically observed when functional viral polymerases have been brought into the cell with infectious viral particles, but newly synthesized viral polymerases at later times after infection remain non-functional (Bloyet et al., 2016). In the presence of SKI-II and ceranib-2, we observed similar kinetics of the inhibition of the synthesis of new viruses in primary PBL. It is not clear if and how ceranib-2 may influence Hsp90 activity; however, it is probably part of the SKI-II-mediated inhibition of MV in PBL. This might be different in BJAB cells, which responded faster (already at day 1) to SKI-II (and ceranib-2).

A number of reports have described ceramide interacting proteins such as the anti-oncogene p53 (Fekry et al., 2018), ceramide activated protein kinase (CAPK) (Huwiler et al., 1996), protein kinase C-ζ (PKCζ) (Wang et al., 2005), cathepsins (Heinrich et al., 1999), and the ribosomal voltage dependent anion channel 2 (VDAC2) (Dadsena et al., 2019), which can induce apoptosis or other types of cell death. Predominantly C18 ceramide has been reported to interact with the SET (I2PP2A) protein and to inhibit its activity, which leads to an activation of the serine/threonine phosphatase PP2A, which dephosphorylates and inactivates the anti-apoptotic protein BCL2 (Dobrowsky et al., 1993; Li et al., 1996; Neviani et al., 2005; Trotta et al., 2007; Mukhopadhyay et al., 2009; Oaks and Ogretmen, 2014). For SKI-II it has been described mainly for cancer cells that this inhibitor can also induce cell death (Leroux et al., 2007; LeBlanc et al., 2015; Yang et al., 2015). Thus, increased ceramide concentrations, as well as decreased S1P concentrations can induce apoptosis or other forms of cell death (Hannun and Obeid, 2008; Young et al., 2013; Oaks and Ogretmen, 2014). In our study, low concentrations of the inhibitors (1 μM SKI-II and 0.5 μM ceranib-2), which induced <5% dead PBL, clearly reduced MV replication. Thus, this suggested that cell death is not responsible for the observed reduction in MV replication.

## Conclusion

In summary, our data indicate that MV replication is reduced by inhibitors of the acid ceramidase and SphKs in primary human PBL and suggest that viral replication is impaired by several mechanisms regulated by sphingolipid-mediated signaling pathways. We can add the aspect that the inhibitors SKI-II and ceranib-2 affect the activities of mTORC1 and Hsp90, and thus reduce MV replication. Future work is required to reveal the exact mechanisms involved and to optimize the use of compounds regulating sphingolipid metabolism in order to inhibit virus replication while maintaining a functional antiviral immune response *in vivo*.

## Data Availability Statement

The datasets generated for this study are available on request to the corresponding author.

## Author Contributions

NB and JS-S: conceptualization. AG, FS, JC, and BK: methodology. JS-S: writing – original draft preparation.

## Conflict of Interest

The authors declare that the research was conducted in the absence of any commercial or financial relationships that could be construed as a potential conflict of interest.
